# The Fungus of Syphilis

**Published:** 1881-04

**Authors:** 


### THE FUNGUS OF SYPHILIS.

---

Dr. Bermann, of Baltimore, has an interesting article on the contagium of syphilis in the *Archives of Medicine.* Upon microscopical examination of absolutely fresh specimens of indurated chancres, "singular collections of micrococci and fungoid growths, firmly adhering to and partly blocking the lumina of most of the lymphatic vessels," were found. The micrococci are principally seen in the lymphatics, where they generally envelop the valves. They are small, strongly refracting bodies, resembling those illustrated by Klebs. The principal changes take place in the lymphatic system, and at some distance from the initial sclerosis, which explains why other investigators have been unsuccessful in discovering these fungoid growths. The walls of the vessels are covered by thick layers of micrococci, the valves of the lymphatics are thickly studded with them, which show by their swollen and hardened condition that they have undergone an inflammatory process. The changes in the lymphatics sometimes consist of an amyloid degeneration of the endothelium and surrounding tissues, destroying the muscle. The micrococci are more prevalent near the original lesion. while some distance from the induration higher developed forms are found. Dr B[illegible]rmann offers the following theory of the disease: Infection takes place by reason of a few micrococci being retained in a lesion of the skin. They are taken up by the lymphatics, when they increase, and by spreading soon obstruct the circulation in them; in consequence an infiltration of the surrounding tissues occurs and an induration is produced. The spores continue to develop; small particles of them enter the blood vessels and are carried into different portions of the body. They take root at those points most favorable for their growth and produce the changes described above. They produce metastasis in various organs, especially in the capillaries of the skin, when by stopping the circulation an extravasation of the blood occurs, which the author thinks is the best possible explanation of the brown spots which remain after the exanthem has disappeared.

We wonder if this discovery will not meet with the same fate as Lostorfer's corpuscles.—*Medical Herald.*

